# Adenin-1-ium hydrogen isophthalate di­methyl­formamide monosolvate

**DOI:** 10.1107/S1600536813034971

**Published:** 2014-01-18

**Authors:** Vandavasi Koteswara Rao, Tausif Siddiqui, Matthias Zeller, Sherri R. Lovelace-Cameron

**Affiliations:** a Department of Chemistry, Youngstown State University, One University, Plaza, Youngstown, OH 44555, USA

## Abstract

In the title proton-transfer organic salt, C_5_H_6.3_N_5_^+^·C_8_H_4.7_O_4_^−^·C_3_H_7_NO, the adeninium moiety is protonated at the N atom in the 1-position of the 6-amino-7*H*-purin-1-ium (adeninium) cation. In the solid state, the second acidic proton of isophthalic acid is partially transferred to the imidazole N atom of the adeninium cation [refined O—H *versus* N—H ratio = 0.70 (11):0.30 (11)]. Through the partially transferred proton, the adeninium cation is strongly hydrogen bonded (N—H⋯O/O—H⋯N) to the isophthalate anion. This strong inter­action is assisted by another N—H⋯O hydrogen bond originating from the adeninium NH_2_ group towards the isophthalate keto O atom, with an *R* ^2^_2_(8) graph-set motif. This arrangement is linked *via* N—H⋯O hydrogen bonds to the O atoms of the carboxyl­ate group of an isophthalate anion. Together, these hydrogen bonds lead to the formation criss-cross zigzag isophthalate⋯adeninium chains lying parallel to (501) and (50-1). The adeninium cations and the isophthalate anions are arranged in infinite π stacks that extend along the *c-*axis direction [inter­planar distance = 3.305 (3) Å]. Mol­ecules are inclined with respect to this direction and within the stacks they are offset by ca. half a mol­ecule each. Combination of the N—H⋯O and O—H⋯N hydrogen bonds with the π–π inter­actions forms infinitely stacked isophthalate⋯adeninium chains, thus leading to a two-dimensional supra­molecular structure with parallel inter­digitating layers formed by the *π* stacked isophthalate⋯adeninium chains. The DMF mol­ecules of crystallization are bonded to the adeninium cations through strong N—H⋯O hydrogen bonds and project into the lattice space in between the anions and cations. There are also C—H⋯O hydrogen bonds present which, combined with the other inter­actions, form a three-dimensional network. The crystal under investigation was found to be split and was handled as if non-merohedrally twinned.

## Related literature   

For supra­molecular structures comprising 3-carb­oxy­benzo­ates, see, for example: Siddiqui *et al.* (2012 ▶). For adenine as a linker and biomolecular building block, see: An *et al.* (2010 ▶). For hydrogen bonding, see: Gilli & Gilli (2009 ▶). For graph-set analysis, see: Etter (1990 ▶); Bernstein *et al.* (1995 ▶)·The crystal under investigation was found to be split and was handled as if non-merohedrally twinned. The orientation matrices for the two components were identified using the program *CELL NOW* (Sheldrick, 2004 ▶).
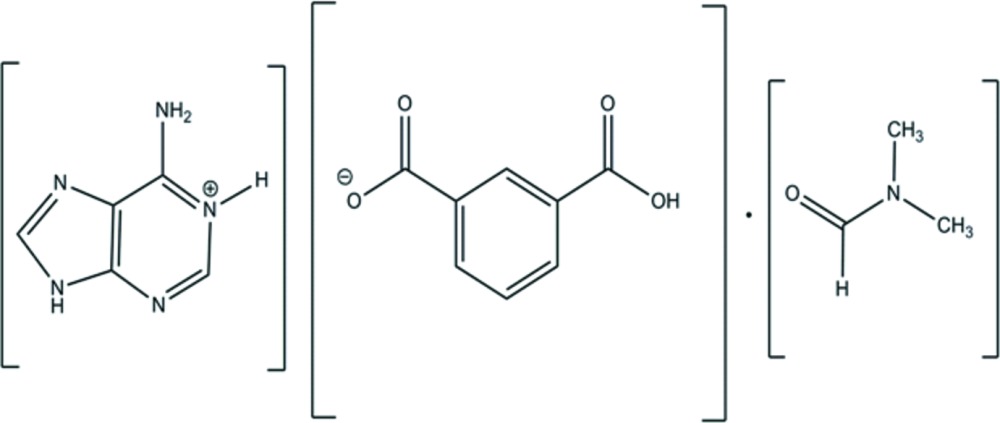


## Experimental   

### 

#### Crystal data  

C_5_H_6.30_N_5_^+^·C_8_H_4.70_O_4_^−^·C_3_H_7_NO
*M* *_r_* = 374.36
Orthorhombic, 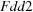

*a* = 38.307 (18) Å
*b* = 46.05 (2) Å
*c* = 3.7832 (18) Å
*V* = 6674 (5) Å^3^
*Z* = 16
Mo *K*α radiation
μ = 0.11 mm^−1^
*T* = 100 K
0.55 × 0.15 × 0.04 mm

#### Data collection  

Bruker SMART APEX CCD diffractometer
Absorption correction: multi-scan (TWINABS; Bruker, 2009 ▶) *T*_min_ = 0.730, *T*_max_ = 1.000
3888 measured reflections
3888 independent reflections
2703 reflections with *I* > 2σ(*I*)

#### Refinement  

*R*[*F* ^2^ > 2σ(*F* ^2^)] = 0.088
*wR*(*F* ^2^) = 0.233
*S* = 1.05
3888 reflections
251 parameters
2 restraints
H atoms treated my a mixture of independent and constrained refinement
Δρ_max_ = 0.58 e Å^−3^
Δρ_min_ = −0.50 e Å^−3^

### 

Data collection: *APEX2* (Bruker, 2011 ▶); cell refinement: *SAINT* (Bruker, 2011 ▶); data reduction: *SAINT*; program(s) used to solve structure: *SHELXS97* (Sheldrick, 2008 ▶); program(s) used to refine structure: *SHELXL2013* (Sheldrick, 2008 ▶) and *SHELXLE* (Hübschle *et al.*, 2011 ▶); molecular graphics: *Mercury* (Macrae *et al.*, 2008 ▶); software used to prepare material for publication: *SHELXL2013* and *publCIF* (Westrip, 2010 ▶).

## Supplementary Material

Crystal structure: contains datablock(s) I, New_Global_Publ_Block. DOI: 10.1107/S1600536813034971/su2677sup1.cif

Structure factors: contains datablock(s) I. DOI: 10.1107/S1600536813034971/su2677Isup2.hkl

Supporting information file. DOI: 10.1107/S1600536813034971/su2677Isup3.cml

CCDC reference:

Additional supporting information:  crystallographic information; 3D view; checkCIF report

## Figures and Tables

**Table 1:** Hydrogen-bond geometry (Å, °)

*D*—H⋯*A*	*D*—H	H⋯*A*	*D*⋯*A*	*D*—H⋯*A*
O2—H2⋯N4	0.84 (2)	1.77 (4)	2.599 (6)	170 (16)
N4—H4*A*⋯O2	0.88	1.75	2.599 (6)	160
N5—H5*B*⋯O1	0.88	2.05	2.913 (7)	168
N1—H1⋯O4^i^	0.88	1.73	2.601 (6)	168
N5—H5*A*⋯O3^i^	0.88	2.07	2.933 (7)	166
N3—H3*A*⋯O5^ii^	0.88	1.84	2.700 (7)	166
C12—H12⋯O5^iii^	0.95	2.27	3.196 (7)	165
C16—H16*C*⋯O1^iv^	0.98	2.63	3.290 (8)	125

Symmetry codes: (i) 
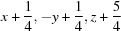
; (ii) 
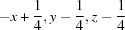
; (iii) 
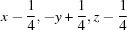
; (iv) 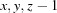.

**Fig. 1.**
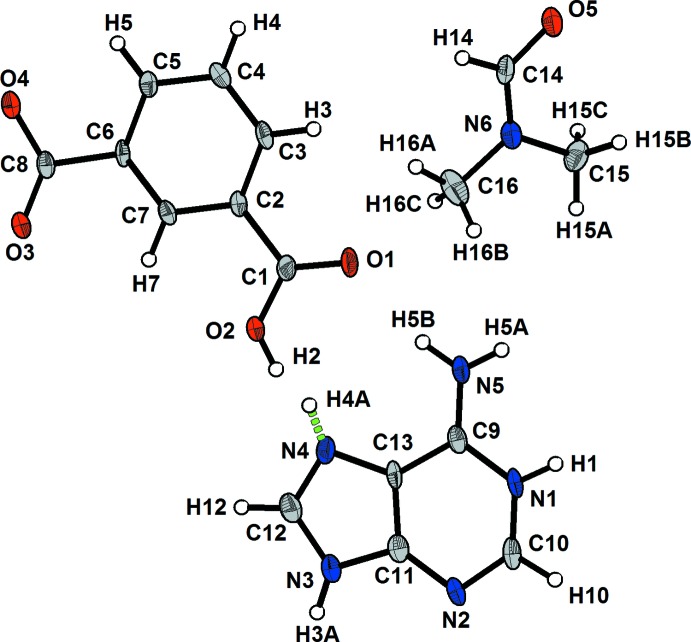
View of the title organic compound, with atom labelling. Displacement ellipsoids are drawn at the 50% probability level. Green dots represent the partially transferred proton to atom N4.

**Fig. 2.**
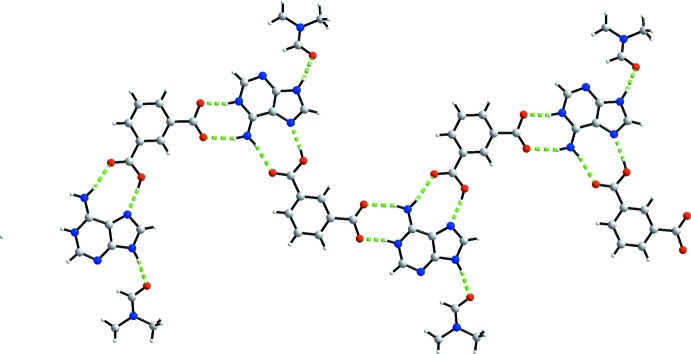
View of one of the one-dimensional undulating chains lying parallel to (501) in the crystal packing of the title compound. Hydrogen bonds are shown as green dashed lines (see Table 1 for details; the minor occupied hydrogen atoms, H4A, have been omitted for clarity).

**Fig. 3.**
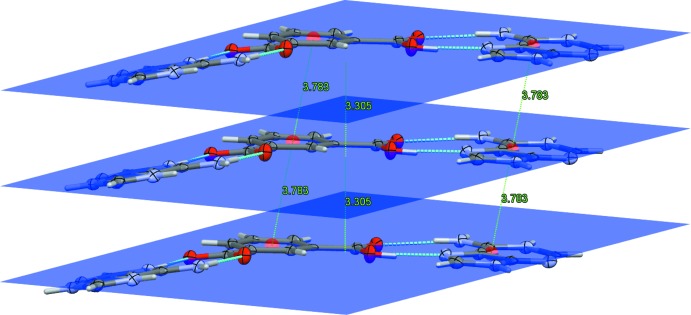
View of the π-stacked chains in the crystal of the title compound. Pink spheres represent centroids of isophthalate benzene rings and adeninium cations; blue planes are weighted averages of each two adeninium and isophthalate ions as displayed. The green dotted lines represent centroid-centroid and interplanar distances. The minor occupied hydrogen atoms, H4A, and the DMF molecules have been omitted for clarity.

## supplementary crystallographic information

### 1. Comment

The title compound was obtained as a result of our studies on magnesium based metal-organic frameworks (MOFs) using solvothermal synthesis. In order to examine the interaction between metal salts and various organic ligands as linkers for possible formation of MOFs, we have extensively tested bis- and tris-carboxylic acid linkers such as isophthalic acid, cyclohexane dicarboxylic acid, and trimesic acid, but also nitrogen based ligands derived from DNA bases such as adenine, which was recently reported as a particularly effective linker and biomolecular building block (An *et al.*, 2010) due to its rigidity and potential multiple coordination modes. We are currently interested in the synthesis of MOFs with Mg, isophthalate and adenine. Reaction of magnesium nitrate with isophthalic acid and adenine at 423 K did, however, not yield the desired extended framework. The presence of multiple Lewis basic sites (amino group) in adenine led to the formation of colourless plate like crystals of the title compound as a minor product along with a sand-like precipitate of Mg formate dihydrate, Mg(HCOO)_2_·2H_2_O.

The asymmetric unit of the title proton-transfer organic salt consists of one adeninium monocation, one isophthalate monoanion, and one molecule of dimethylformamide (DMF) [Fig. 1]. The adeninium moiety is protonated at the nitrogen in the 1-position of the 6-amino-7*H*-purin-1-ium (adeninium) cation. In the solid state the second acidic proton of isophthalic acid, H2, is partially transferred to the imidazole nitrogen (N4) of the adeninium cation[(refined O—H *versus* N—H ratio = 0.70 (11):0.30 (11)]. Through the partially transferred proton the adeninium cation is strongly hydrogen bonded to the isophthalate anion (Gilli *et al.*, 2009). This strong interaction is assisted by another N—H···O hydrogen bond originating from the adeninium NH_2_ group towards the isophthalate keto oxygen atom, O1, forming an *R*^2^_2_(8) graph set motif (Etter, 1990; Bernstein *et al.*, 1995). This arrangement is linked via N-H···O hydrogen bonds to the O atoms of the carboxylate group of the isophthalate anion. Combined these hydrogen bonds lead to the formation of one-dimensional zigzag isophthalate···adeninium chains that lie parallel to planes (501) and (50-1). The various hydrogen bonds are given in Table 1.

Mono-deprotonated isophthalate anions have also been observed in other related compounds, such as for dimethylammonium 3-carboxybenzoate (Siddiqui *et al.*, 2012). In that structure the isophthalate monoanions are hydrogen bonded with neighbouring isophthalates as well as dimethylammonium cations resulting in the formation of a double-chain-like structure. In the title compound, the isophthalate monoanions are hydrogen-bonded with adeninium cations which leads to the formation of one-dimensional undulated isophthalate···adeninium chains. The adeninium cations and the isophthalate monoanions, from the zigzag chains, are in turn arranged in infinite *π* stacks that extend along the direction of the *c* axis [interplanar distances 3.305 (3) Å]. Molecules are inclined with respect to this direction and within the stacks they are offset by ca. half a molecule each, 1.736 (3) Å for the isophthalate anions and 1.827 (3) Å for the adeninium cations. The average centroid-centroid distances are thus substantially larger than the interplanar distances; 3.783 (4) Å for both the adeninium cations and the isophthalate anions. For the isophthalate anions, *π-π* stacking is thus mostly between the phenyl ring of one molecule and the carboxylate group of another. In the adeninium cations *π-π* stacking interactions are between the centers of the pyrimidine and imidazole rings.

Combination of the N—H···O and O—H···N hydrogen bonds with the *π-π* interactions forms infinitely stacked isophthalate···adeninium chains (Table 1 and Fig. 2), thus leading to a two-dimensional supramolecular structure with parallel interdigitating layers formed by the *π* stacked isophthalate···adeninium chains (Fig. 3). The DMF molecules are bonded to the adeninium cation through strong N—H···O hydrogen bonds and are projecting into the lattice space in between the anions and cations (Table 1). There are also C-H···O hydrogen bonds present (Table 1) which together with the other interactions form a three-dimensional network.

### 2. Experimental

The compound was synthesized under solvothermal conditions. In a typical synthesis, Mg(NO_3_)_2_·6H_2_O (0.128 g, 0.5 mmol) and adenine (0.068 g, 0.5 mmol) were dissolved in DMF (10.0 ml). Then isophthalic acid (0.166 g, 0.5 mmol) was added to the reaction mixture under continuous stirring. The mixture was stirred for 30 minutes before transferring the mixture into a 23 ml teflon-lined stainless steel autoclave. The final mixture, with a composition of 1:1:1, was heated to 423 K for 96 h. The autoclave was then cooled to room temperature, yielding colourless plate-like crystals of the title compound as a minor product, along with a sand like precipitate of Mg formate dihydrate, Mg(HCOO)_2_·2(H_2_O), (ICSD #151330).

### 3. Refinement

The crystal under investigation was found to be split and was handled as if non-merohedrally twinned. The orientation matrices for the two components were identified using the program Cell Now (Sheldrick, 2004), which reports the second moiety to be related to the first by a 2.8 degree rotation around either the reciprocal *a*-axis, or around the real axis 1 0 - 0.6. The two components were integrated using Saint, resulting in a total of 22391 reflections. 6713 reflections (1732 unique) involved component 1 only (mean I/σ = 3.8), 6662 reflections (1736 unique) involved component 2 only (mean I/σ = 2.5), and 9016 reflections (3181 unique) involved both components (mean I/σ = 5.2). The exact twin matrix identified by the integration program was found to be -1.00000 - 0.00215 - 0.00999, -0.00315 0.99902 0.53675, 0.00009 0.00362 - 0.99903.

The data were corrected for absorption using TWINABS (Bruker, 2009), and the structure was solved using direct methods with only the non-overlapping reflections of component 1. The structure was refined using the hklf 5 routine with all reflections of component 1 (including the overlapping ones), resulting in a BASF value of 0.317 (5). In the absence of significant anomalous scatterers Friedel pairs were merged during correction for absorption effects with TWINABS.

The *R*_int_ value given is for all reflections and is based on agreement between observed single and composite intensities and those calculated from refined unique intensities and twin fractions (TWINABS; Bruker, 2009).

Hydrogen atoms were placed in calculated positions with C—H = 0.95 Å (aromatic H), 0.99 Å (methyl H) or 0.88 Å (N—H). Methyl group H atoms were allowed to rotate freely around the C—C bond to best fit the experimental electron density. Carboxylic acid hydrogen atoms were located in difference electron density maps, but were placed in calculated positions with fixed C—O—H angles, but with the C—C—O—H dihedral angles and the O—H distances refined with the constraint AFIX 148 (Sheldrick, 2008). *U*_iso_(H) = 1.5U_eq_(C/O) for methyl and carboxylic acid H atoms, and = 1.2U_eq_(C/N) for aromatic and adenine H-atoms. One of the acidic hydrogen atoms was refined as disordered over a carboxylate *versus* a nitrogen bound site. The occupancy rate for the major O-bound site refined to 0.70 (9).

### Crystal data

**Table:**

C_5_H_6.30_N_5_^+^·C_8_H_4.70_O_4_^−^·C_3_H_7_NO	*D*_x_ = 1.490 Mg m^−^^3^
*M**_r_* = 374.36	Mo *K*α radiation, λ = 0.71073 Å
Orthorhombic, *F**d**d*2	Cell parameters from 290 reflections
*a* = 38.307 (18) Å	θ = 4.3–30.4°
*b* = 46.05 (2) Å	µ = 0.11 mm^−^^1^
*c* = 3.7832 (18) Å	*T* = 100 K
*V* = 6674 (5) Å^3^	Plate, colourless
*Z* = 16	0.55 × 0.15 × 0.04 mm
*F*(000) = 3136	

### Data collection

**Table:**

Bruker SMART APEX CCD diffractometer	3888 independent reflections
Radiation source: fine-focus sealed tube	2703 reflections with *I* > 2σ(*I*)
Graphite monochromator	θ_max_ = 30.6°, θ_min_ = 1.4°
ω scans	*h* = 0→54
Absorption correction: multi-scan (TWINABS; Bruker, 2009)	*k* = 0→66
*T*_min_ = 0.730, *T*_max_ = 1.000	*l* = 0→5
3888 measured reflections	

### Refinement

**Table:**

Refinement on *F*^2^	Primary atom site location: structure-invariant direct methods
Least-squares matrix: full	Secondary atom site location: difference Fourier map
*R*[*F*^2^ > 2σ(*F*^2^)] = 0.088	Hydrogen site location: mixed
*wR*(*F*^2^) = 0.233	H atoms treated by a mixture of independent and constrained refinement
*S* = 1.05	*w* = 1/[σ^2^(*F*_o_^2^) + (0.0928*P*)^2^ + 48.0632*P*] where *P* = (*F*_o_^2^ + 2*F*_c_^2^)/3
3888 reflections	(Δ/σ)_max_ < 0.001
251 parameters	Δρ_max_ = 0.58 e Å^−^^3^
2 restraints	Δρ_min_ = −0.50 e Å^−^^3^

### Special details

**Table:**

Geometry. All e.s.d.'s (except the e.s.d. in the dihedral angle between two l.s. planes) are estimated using the full covariance matrix. The cell e.s.d.'s are taken into account individually in the estimation of e.s.d.'s in distances, angles and torsion angles; correlations between e.s.d.'s in cell parameters are only used when they are defined by crystal symmetry. An approximate (isotropic) treatment of cell e.s.d.'s is used for estimating e.s.d.'s involving l.s. planes.
Refinement. Refined as a 2-component twin.

### Fractional atomic coordinates and isotropic or equivalent isotropic displacement parameters (Å^2^)

**Table:**

	*x*	*y*	*z*	*U*_iso_*/*U*_eq_	Occ. (<1)
C1	0.05925 (14)	0.13091 (12)	0.512 (2)	0.0190 (13)	
C2	0.04322 (13)	0.15984 (12)	0.4520 (16)	0.0123 (11)	
C3	0.06001 (14)	0.18481 (13)	0.566 (2)	0.0194 (13)	
H3	0.0822	0.1834	0.6767	0.023*	
C4	0.04487 (14)	0.21170 (12)	0.5214 (17)	0.0156 (11)	
H4	0.0564	0.2287	0.6040	0.019*	
C5	0.01246 (13)	0.21387 (12)	0.3538 (18)	0.0161 (12)	
H5	0.0019	0.2323	0.3205	0.019*	
C6	−0.00425 (13)	0.18877 (12)	0.2360 (18)	0.0144 (11)	
C7	0.01070 (13)	0.16197 (12)	0.2957 (19)	0.0157 (12)	
H7	−0.0014	0.1448	0.2289	0.019*	
C8	−0.03904 (14)	0.19030 (12)	0.0547 (19)	0.0171 (12)	
C9	0.12077 (13)	0.05012 (13)	0.7880 (19)	0.0175 (12)	
C10	0.13647 (13)	0.00114 (14)	0.7890 (18)	0.0181 (12)	
H10	0.1535	−0.0127	0.8607	0.022*	
C11	0.08680 (14)	0.01200 (13)	0.5326 (19)	0.0183 (12)	
C12	0.04224 (15)	0.03628 (13)	0.335 (2)	0.0230 (14)	
H12	0.0206	0.0404	0.2233	0.028*	
C13	0.09038 (13)	0.04118 (13)	0.604 (2)	0.0185 (13)	
C14	0.18697 (14)	0.19317 (13)	0.289 (2)	0.0216 (14)	
H14	0.1657	0.2008	0.3782	0.026*	
C15	0.21815 (16)	0.15444 (15)	0.010 (2)	0.0275 (15)	
H15A	0.2141	0.1338	−0.0415	0.041*	
H15B	0.2368	0.1563	0.1853	0.041*	
H15C	0.2250	0.1645	−0.2079	0.041*	
C16	0.15440 (17)	0.15073 (17)	0.128 (3)	0.0343 (17)	
H16A	0.1355	0.1615	0.2439	0.051*	
H16B	0.1577	0.1321	0.2481	0.051*	
H16C	0.1483	0.1473	−0.1198	0.051*	
N1	0.14293 (12)	0.02850 (11)	0.8754 (17)	0.0188 (11)	
H1	0.1621	0.0327	0.9926	0.023*	
N2	0.10912 (11)	−0.00944 (11)	0.6145 (17)	0.0194 (11)	
N3	0.05564 (12)	0.00939 (11)	0.3618 (16)	0.0184 (11)	
H3A	0.0461	−0.0068	0.2839	0.022*	
N4	0.06190 (12)	0.05623 (11)	0.4781 (17)	0.0210 (12)	
H4A	0.0579	0.0750	0.4911	0.025*	0.30 (11)
N5	0.12746 (11)	0.07718 (11)	0.8667 (17)	0.0195 (11)	
H5A	0.1468	0.0817	0.9795	0.023*	
H5B	0.1126	0.0909	0.8070	0.023*	
N6	0.18633 (12)	0.16733 (11)	0.1475 (19)	0.0219 (12)	
O1	0.08756 (10)	0.12818 (9)	0.6562 (15)	0.0227 (10)	
O2	0.04090 (10)	0.10943 (9)	0.3921 (16)	0.0239 (11)	
H2	0.047 (3)	0.0918 (8)	0.40 (4)	0.036*	0.70 (11)
O3	−0.05527 (10)	0.16840 (9)	−0.0170 (16)	0.0247 (11)	
O4	−0.04896 (10)	0.21663 (9)	−0.0238 (14)	0.0219 (10)	
O5	0.21343 (10)	0.20846 (9)	0.3152 (15)	0.0233 (10)	

### Atomic displacement parameters (Å^2^)

**Table:**

	*U*^11^	*U*^22^	*U*^33^	*U*^12^	*U*^13^	*U*^23^
C1	0.011 (2)	0.022 (2)	0.024 (4)	0.0015 (19)	0.005 (3)	0.002 (3)
C2	0.009 (2)	0.022 (2)	0.006 (3)	0.0007 (19)	0.002 (2)	0.000 (2)
C3	0.011 (2)	0.029 (3)	0.019 (3)	−0.002 (2)	−0.006 (3)	0.000 (3)
C4	0.014 (2)	0.025 (3)	0.008 (3)	−0.006 (2)	0.001 (2)	−0.001 (3)
C5	0.011 (2)	0.023 (3)	0.014 (3)	0.002 (2)	0.000 (2)	−0.002 (3)
C6	0.008 (2)	0.025 (3)	0.011 (3)	0.0014 (19)	−0.002 (2)	0.003 (3)
C7	0.010 (2)	0.022 (2)	0.016 (3)	−0.0029 (19)	0.001 (2)	0.002 (3)
C8	0.010 (2)	0.025 (3)	0.016 (3)	0.001 (2)	0.003 (2)	0.001 (3)
C9	0.011 (2)	0.028 (3)	0.013 (3)	−0.001 (2)	0.002 (2)	0.001 (3)
C10	0.009 (2)	0.036 (3)	0.009 (3)	0.002 (2)	0.006 (2)	0.003 (3)
C11	0.013 (2)	0.028 (3)	0.014 (3)	0.000 (2)	0.005 (2)	0.001 (3)
C12	0.015 (2)	0.031 (3)	0.023 (4)	−0.003 (2)	0.002 (3)	0.003 (3)
C13	0.007 (2)	0.028 (3)	0.020 (3)	0.001 (2)	0.001 (2)	−0.002 (3)
C14	0.012 (2)	0.028 (3)	0.025 (4)	0.005 (2)	0.000 (3)	0.003 (3)
C15	0.024 (3)	0.032 (3)	0.026 (4)	0.010 (2)	−0.002 (3)	−0.006 (3)
C16	0.023 (3)	0.050 (4)	0.030 (4)	−0.009 (3)	−0.001 (3)	−0.008 (4)
N1	0.0079 (18)	0.031 (2)	0.018 (3)	−0.0006 (18)	−0.003 (2)	−0.001 (3)
N2	0.010 (2)	0.029 (2)	0.019 (3)	−0.0032 (18)	−0.001 (2)	−0.002 (3)
N3	0.013 (2)	0.028 (2)	0.015 (3)	−0.0002 (18)	−0.001 (2)	0.000 (2)
N4	0.012 (2)	0.025 (2)	0.026 (3)	0.0035 (18)	0.002 (2)	0.002 (3)
N5	0.0090 (19)	0.026 (2)	0.023 (3)	0.0006 (18)	0.000 (2)	−0.001 (3)
N6	0.015 (2)	0.027 (2)	0.024 (3)	0.0008 (18)	−0.001 (2)	−0.001 (3)
O1	0.0114 (18)	0.030 (2)	0.027 (3)	0.0020 (16)	−0.0071 (19)	0.000 (2)
O2	0.0124 (17)	0.0213 (19)	0.038 (3)	−0.0002 (15)	−0.008 (2)	0.000 (2)
O3	0.0128 (17)	0.025 (2)	0.037 (3)	−0.0015 (15)	−0.006 (2)	0.000 (2)
O4	0.0119 (17)	0.026 (2)	0.028 (3)	0.0003 (16)	−0.002 (2)	0.000 (2)
O5	0.0141 (18)	0.030 (2)	0.025 (3)	0.0009 (16)	−0.002 (2)	−0.003 (2)

### Geometric parameters (Å, º)

**Table:**

C1—O1	1.220 (7)	C11—N3	1.363 (8)
C1—O2	1.296 (7)	C11—C13	1.378 (8)
C1—C2	1.485 (7)	C12—N4	1.305 (8)
C2—C7	1.382 (8)	C12—N3	1.345 (8)
C2—C3	1.386 (8)	C12—H12	0.9500
C3—C4	1.378 (8)	C13—N4	1.378 (7)
C3—H3	0.9500	C14—O5	1.238 (7)
C4—C5	1.398 (8)	C14—N6	1.305 (8)
C4—H4	0.9500	C14—H14	0.9500
C5—C6	1.394 (8)	C15—N6	1.453 (8)
C5—H5	0.9500	C15—H15A	0.9800
C6—C7	1.380 (7)	C15—H15B	0.9800
C6—C8	1.501 (8)	C15—H15C	0.9800
C7—H7	0.9500	C16—N6	1.444 (8)
C8—O3	1.215 (7)	C16—H16A	0.9800
C8—O4	1.305 (7)	C16—H16B	0.9800
C9—N5	1.307 (7)	C16—H16C	0.9800
C9—N1	1.349 (7)	N1—H1	0.8800
C9—C13	1.417 (8)	N3—H3A	0.8800
C10—N1	1.325 (8)	N4—H4A	0.8800
C10—N2	1.331 (8)	N5—H5A	0.8800
C10—H10	0.9500	N5—H5B	0.8800
C11—N2	1.342 (7)	O2—H2	0.84 (2)
			
O1—C1—O2	124.1 (5)	N4—C13—C11	110.1 (5)
O1—C1—C2	121.9 (5)	N4—C13—C9	132.4 (5)
O2—C1—C2	114.0 (5)	C11—C13—C9	117.5 (5)
C7—C2—C3	119.5 (5)	O5—C14—N6	124.5 (6)
C7—C2—C1	120.1 (5)	O5—C14—H14	117.7
C3—C2—C1	120.3 (5)	N6—C14—H14	117.7
C4—C3—C2	120.8 (5)	N6—C15—H15A	109.5
C4—C3—H3	119.6	N6—C15—H15B	109.5
C2—C3—H3	119.6	H15A—C15—H15B	109.5
C3—C4—C5	119.6 (5)	N6—C15—H15C	109.5
C3—C4—H4	120.2	H15A—C15—H15C	109.5
C5—C4—H4	120.2	H15B—C15—H15C	109.5
C6—C5—C4	119.6 (5)	N6—C16—H16A	109.5
C6—C5—H5	120.2	N6—C16—H16B	109.5
C4—C5—H5	120.2	H16A—C16—H16B	109.5
C7—C6—C5	119.9 (5)	N6—C16—H16C	109.5
C7—C6—C8	119.1 (5)	H16A—C16—H16C	109.5
C5—C6—C8	121.0 (5)	H16B—C16—H16C	109.5
C6—C7—C2	120.5 (5)	C10—N1—C9	121.6 (5)
C6—C7—H7	119.8	C10—N1—H1	119.2
C2—C7—H7	119.8	C9—N1—H1	119.2
O3—C8—O4	124.9 (6)	C10—N2—C11	110.3 (5)
O3—C8—C6	121.1 (5)	C12—N3—C11	106.8 (5)
O4—C8—C6	114.0 (5)	C12—N3—H3A	126.6
N5—C9—N1	121.6 (5)	C11—N3—H3A	126.6
N5—C9—C13	123.4 (5)	C12—N4—C13	104.3 (5)
N1—C9—C13	115.0 (5)	C12—N4—H4A	127.9
N1—C10—N2	128.1 (6)	C13—N4—H4A	127.9
N1—C10—H10	115.9	C9—N5—H5A	120.0
N2—C10—H10	115.9	C9—N5—H5B	120.0
N2—C11—N3	127.0 (6)	H5A—N5—H5B	120.0
N2—C11—C13	127.5 (6)	C14—N6—C16	121.3 (6)
N3—C11—C13	105.5 (5)	C14—N6—C15	120.3 (5)
N4—C12—N3	113.4 (6)	C16—N6—C15	118.4 (6)
N4—C12—H12	123.3	C1—O2—H2	126 (8)
N3—C12—H12	123.3		
			
O1—C1—C2—C7	−178.4 (7)	N2—C11—C13—C9	−0.6 (11)
O2—C1—C2—C7	3.1 (9)	N3—C11—C13—C9	179.1 (6)
O1—C1—C2—C3	−1.4 (10)	N5—C9—C13—N4	−1.9 (12)
O2—C1—C2—C3	−179.9 (6)	N1—C9—C13—N4	178.6 (7)
C7—C2—C3—C4	−0.9 (10)	N5—C9—C13—C11	178.9 (6)
C1—C2—C3—C4	−178.0 (6)	N1—C9—C13—C11	−0.5 (9)
C2—C3—C4—C5	−1.0 (10)	N2—C10—N1—C9	−0.1 (11)
C3—C4—C5—C6	0.4 (9)	N5—C9—N1—C10	−178.6 (6)
C4—C5—C6—C7	2.2 (9)	C13—C9—N1—C10	0.9 (9)
C4—C5—C6—C8	−179.9 (6)	N1—C10—N2—C11	−0.9 (10)
C5—C6—C7—C2	−4.1 (9)	N3—C11—N2—C10	−178.4 (7)
C8—C6—C7—C2	177.9 (6)	C13—C11—N2—C10	1.2 (10)
C3—C2—C7—C6	3.5 (9)	N4—C12—N3—C11	−0.7 (8)
C1—C2—C7—C6	−179.5 (6)	N2—C11—N3—C12	−179.7 (7)
C7—C6—C8—O3	5.3 (10)	C13—C11—N3—C12	0.5 (7)
C5—C6—C8—O3	−172.6 (7)	N3—C12—N4—C13	0.6 (8)
C7—C6—C8—O4	−172.8 (6)	C11—C13—N4—C12	−0.2 (8)
C5—C6—C8—O4	9.3 (9)	C9—C13—N4—C12	−179.4 (8)
N2—C11—C13—N4	−179.9 (6)	O5—C14—N6—C16	179.7 (7)
N3—C11—C13—N4	−0.2 (7)	O5—C14—N6—C15	0.6 (11)

### Hydrogen-bond geometry (Å, º)

**Table:**

*D*—H···*A*	*D*—H	H···*A*	*D*···*A*	*D*—H···*A*
O2—H2···N4	0.84 (2)	1.77 (4)	2.599 (6)	170 (16)
N4—H4*A*···O2	0.88	1.75	2.599 (6)	160
N5—H5*B*···O1	0.88	2.05	2.913 (7)	168
N1—H1···O4^i^	0.88	1.73	2.601 (6)	168
N5—H5*A*···O3^i^	0.88	2.07	2.933 (7)	166
N3—H3*A*···O5^ii^	0.88	1.84	2.700 (7)	166
C12—H12···O5^iii^	0.95	2.27	3.196 (7)	165
C16—H16*C*···O1^iv^	0.98	2.63	3.290 (8)	125

Symmetry codes: (i) *x*+1/4, −*y*+1/4, *z*+5/4; (ii) −*x*+1/4, *y*−1/4, *z*−1/4; (iii) *x*−1/4, −*y*+1/4, *z*−1/4; (iv) *x*, *y*, *z*−1.
